# Impact de la pandémie COVID-19 sur la vaccination des enfants au Maroc: enquête électronique auprès de 103 pédiatres

**DOI:** 10.11604/pamj.2021.38.134.24104

**Published:** 2021-02-05

**Authors:** Nabila Chekhlabi, Raja Arrab, Said Ettair, Nouzha Dini

**Affiliations:** 1 Service de Pédiatrie, Hôpital International Universitaire Cheikh Khalifa Ibn Zayed, BP 82403 Oum Rabii, Hay Hassani Boulevard Mohamed Taieb Naciri, Casablanca, Maroc,; 2 Université Mohammed VI des Sciences de la Santé UM6SS, Anfa City, Boulevard Mohammed Taïeb Naciri, Hay Hassani 82 403 Casablanca, Maroc,; 3 Faculté de Médecine de Rabat, Université Mohammed V, Rabat, Maroc

**Keywords:** Vaccination, enfant, confinement, COVID-19, Vaccination, child, containment, COVID-19

## Abstract

**Introduction:**

nul ne peut nier les résultats protecteurs satisfaisants de la vaccination contre plusieurs maladies graves dans le monde et particulièrement au Maroc. L´extension de la pandémie COVID-19 dans notre pays a entrainé une baisse importante de la vaccination des enfants, ce qui pourrait causer des flambées épidémiques dans le futur. D´où l´intervention du ministère de la santé pour pallier à cette situation. L´objectif était de montrer l´ampleur du relâchement de la vaccination en temps COVID-19 et sortir avec des recommandations visant à rétablir ce changement.

**Méthodes:**

il s´agit d´une étude transversale sur l´impact de cette pandémie sur le suivi vaccinal des enfants. Nous avons mené une enquête nationale auprès des pédiatres sous forme d´un questionnaire électronique via Google Forms. Nous avons procédé à la collecte, l´analyse et l´interprétation des résultats.

**Résultats:**

cent trois pédiatres marocains ont répondu au questionnaire. Plus de 2 tiers des pédiatres exercent au secteur privé et pratiquent tout le calendrier vaccinal. La majorité des pédiatres (95%) ont reçu des questions émanant des craintes des parents sur la vaccination. Nous avons noté que 82,5% des parents hésitent à venir au cabinet et 5,8% refusent de faire la vaccination pendant cette période. Environ 22% des pédiatres ont arrêté totalement la vaccination et 72,8% ont décalé les vaccins d´un mois.

**Conclusion:**

il est essentiel de maintenir la confiance de la population dans la vaccination. Une évaluation continue de la couverture vaccinale, des recommandations claires et une large sensibilisation du grand public seront nécessaire pour répondre aux changements vaccinaux durant la pandémie de COVID-19.

## Abstract (en)

**Introduction:**

no one can deny that vaccination against several serious diseases in the world, and particularly in Morocco, has given very satisfactory protective results. The extension of the COVID-19 pandemic in our country has led to a significant decline in childhood immunization, which could have severe repercussions increasing the risk of future outbreaks. Hence, the measures of the Ministry of Health to correct the situation. The purpose of this study was to highlight the extent of vaccine release during COVID-19 pandemic and to make recommendations to restore vaccination programmes.

**Methods:**

we conducted a cross-sectional study of the effect of containment measures during this pandemic on the monitoring of children´s vaccinations. We conducted a national survey of pediatricians using an electronic questionnaire administered via Google Forms. We collected, analyzed and interpreted the results.

**Results:**

one hundred and three Moroccan pediatricians answered the questionnaire. More than 2-thirds (78.6%) of pediatricians practiced in the private sector and delivered vaccines in the immunization schedule. The majority of pediatricians (95%) were asked about parental vaccine concerns. We noted that 82.5% of parents were reluctant to go to the local health department and 5.8% refused to take vaccination during COVID-19 pandemic. About 22% of pediatricians completely stopped immunization services and 72.8% delayed immunizations from 3 to 4 weeks. Vaccination stoppage involved older children in two thirds of cases.

**Conclusion:**

it is essential to maintain public confidence in vaccination. Ongoing and timely assessment of vaccine coverage as well as clear recommendations and broad public awareness are essential to respond to vaccine changes during the COVID-19 pandemic.

## Introduction

La vaccination est la pierre angulaire de la prévention. C´est un service de santé essentiel qui protège les enfants et les sujets sensibles contre les maladies infectieuses invasives. La vaccination permet aux individus et aux communautés de rester protégés et diminue la probabilité d´une poussée épidémique. Au Maroc, grâce au programme national d´immunisation du ministère de la santé et aux efforts de la pédiatrie libérale, certaines maladies ont pu être éradiquées avec des taux de couverture vaccinale obtenus avoisinant les 95% [1]. Depuis le 10 mars 2020, la COVID-19 est déclarée, par l´OMS, comme une pandémie mondiale avec installation obligatoire, dans plusieurs pays, d´interventions préventives rigoureuses telles que le confinement à domicile, la distanciation sociale, la fermeture d´écoles, le port obligatoire de masques et le dépistage des contacts [2]. De telles mesures ont entraîné une diminution de l´accessibilité aux services de vaccination de routine, ce qui a provoqué une baisse significative du taux de vaccination. L´arrêt des vaccinations des enfants de moins de 18 mois pendant cette période aurait certainement des conséquences redoutables en termes de morbidité et de mortalité, avec possibilité d´éclosion de foyers de maladies contagieuses longtemps prévenues par la vaccination. D´où l´idée de notre enquête. Ce questionnaire est un outil électronique permettant d´évaluer directement et de la façon la plus large possible l´effet négatif de la pandémie COVID-19 sur la vaccination des enfants au Maroc. Il s´agit de s´interroger sur les craintes des parents et les attitudes vaccinales des pédiatres au cours de cette période critique. Nous avons remarqué une baisse importante de taux de vaccination des enfants aux centres de santé et aux cabinets pédiatriques au cours du confinement [1]. Beaucoup de parents ont raté la vaccination de leurs enfants et même certains pédiatres ont retardé les rendez-vous des vaccinations en l´absence de recommandations nationales précoces. Actuellement, la pandémie COVID-19 est le sujet de discussion mondial parmi le grand public. La progression rapide de cette incontrôlable et contagieuse infection a suscité une grande panique avec des changements dans le mode vie et une réduction de la mobilité des individus volontairement ou imposée par les autorités dans le cadre du confinement sanitaire. Les conséquences immédiates de ces mesures sont la mise en mode pause de l´activité de la plupart des secteurs notamment celui de la santé qui s´est organisé essentiellement dans la prise en charge des patients atteints de COVID-19. Nous nous sommes donc intéressés à évaluer: l´impact négatif du confinement en temps COVID-19 sur la vaccination; la perception des professionnels de santé, particulièrement les pédiatres sur ce sujet.

## Méthodes

Nous avons mené une étude transversale sous forme d´une enquête électronique auprès des pédiatres du Maroc. L´outil de l´enquête est un questionnaire électronique élaboré par notre équipe pédiatrique. Il contenait 18 questions et prenait environ 5 minutes à remplir. Le questionnaire a été conçu à partir des items émanant des questions posées par les parents et les pédiatres. Il a été divisé en deux parties à savoir **une page d´accueil informant le médecin:** des objectifs de l´enquête ; de l´anonymat et de la confidentialité des réponses données ; et la procédure à suivre pour répondre aux questions. **Puis il y a 18 questions:** soit 9 questions générales sur les pédiatres et la pratique vaccinale puis 9 questions sur la problématique d´arrêt de la vaccination au cours de la pandémie COVID 19. Nous avons choisi des questions à choix multiples à la place des questions ouvertes pour que ça soit plus clair et plus facile à répondre. **L´évaluation de l´impact de la pandémie sur la vaccination** a été effectuée à l´aide de questions portant sur les éléments suivants : doutes et comportements des parents, attitude des pédiatres concernant la vaccination au début puis en pleine pandémie. L´âge des enfants intéressés par le retard. **Les différentes étapes de l´enquête:** élaboration du questionnaire; mise en place de ce questionnaire en ligne à l´aide de la plateforme Google Forms; mettre le questionnaire à la portée des participants à travers: leurs adresse email ; des groupes WhatsApp de pédiatres; recueil des données à travers le logiciel SPSS ; analyses des résultats: les données ont été analysées et résumées à l´aide de statistiques descriptives et les résultats ont été affichés dans des graphiques; synthèse de l´enquête. **Les participants:** les pédiatres de secteur privé ou public, une dizaine de villes (Casablanca, Mohammadia, Benslimane, Rabat, El-Jadida, Beni Mellal, Marrakech, Fès, Agadir, Tanger, Boujdour, Laayoune).

## Résultats

**Informations générales:** un total de 103 pédiatres a répondu au questionnaire. Les pédiatres de sexe féminin représentent 82,5% soit un sexe ratio de 0,2. L´âge moyen des participants est de 39 ans avec des extrêmes de 31 et 64 ans. La durée moyenne d´exercice est de 8 ans et demi [extrêmes de 1 an et 35 ans]. Un nombre de 81 pédiatres [soit 78,6%] exercent au secteur libéral alors que 22 pédiatres [21,4%] travaillent aux hôpitaux publics. La majorité des participants [72 pédiatres soit 70%] exercent dans la grande ville économique de Casablanca, le reste sont répartis sur 13 autres villes. La plupart des pédiatres [soit 84,5%] font la vaccination dans leur pratique courante, parmi eux, 92,3% font tout le calendrier vaccinal alors que 7,7% font seulement les vaccins complémentaires du programme national d´immunisation.

**Impact de la pandémie COVID 19 sur la vaccination: craintes des parents:** 98 pédiatres [soit 95%] ont reçu des appels téléphoniques et/ou des questions sur les réseaux sociaux de la part des parents sur la vaccination.

**Figure 1:**
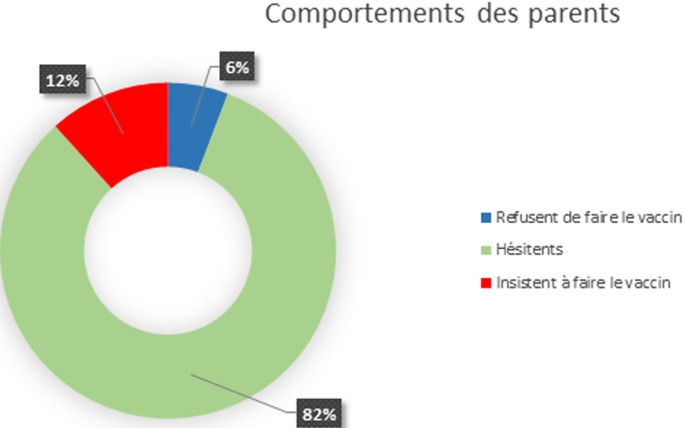
graphique en anneau montrant le comportement des parents vis-à-vis de la vaccination de leurs enfants

**Attitudes des parents:** 82,5% des parents étaient hésitants à venir au cabinet pour faire le vaccin, 6% ont refusé de faire le vaccin pendant la période de COVID 19 et 11,5% des parents, par contre, ont insisté à faire le vaccin à temps (Figure 1). Les raisons de ce comportement étaient les craintes des parents quant à l´exposition potentielle de leurs enfants au COVID-19 lors de visites des cabinets dans 68%, les difficultés d´accès liées au confinement dans 17% ainsi que les rumeurs circulant sur l´influence négative des vaccins sur l´immunité anti virale dans 15%.

**Figure 2:**
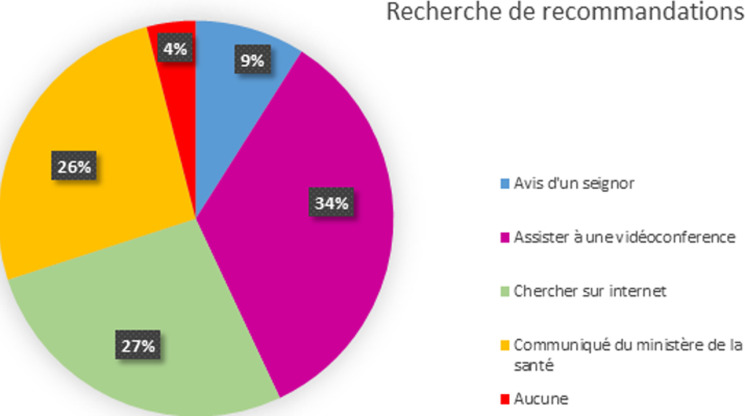
graphique en secteur 3D montrant les différents moyens que les pédiatres ont utilisé pour recherche d´informations sur la vaccination au cours de COVID 19 que les pédiatres

**Attitudes des pédiatres pendant la pandémie COVID 19:** au cours du premier mois de la pandémie [mars 2020], et en absence de recommandations claires ministérielles ou de la part des sociétés savantes : 21,4% des pédiatres ont arrêté complétement la vaccination et 72,8% ont décalé les vaccins de 3 à 4 semaines, alors que le reste n´ont fait aucun changement. Cet arrêt ou décalage des vaccins a intéressé dans 70,9% l´enfant, dans 16,5% le nourrisson de 6 à 18 mois et seulement dans 5,8% les moins de 6 mois. Durant le 2^e^ et le 3^e^ mois de la pandémie, 40% des pédiatres ont gardé la même attitude, alors qu´environ 60% ont changé de stratégie vaccinale. Ces derniers ont affirmé avoir sensibilisé les parents de l´importance du suivi vaccinal et ont entamé un rattrapage rapide des vaccins ratés. Sources d´informations : Nous avons demandé aux pédiatres l´origine de documentation et de recommandations sur la vaccination des enfants au cours de COVID (Figure 2: assister à des vidéo-conférence sur ce thème: 34%; chercher sur internet des recommandations de société savante: 27,2%; se fier au communiqué ministériel sur la vaccination : 26,6%; demander l´avis d´un professeur pédiatrique: 8,7%.

## Discussion

Selon les différentes publications, la COVID-19 semble infecter moins fréquemment l´enfant et provoquer des symptômes plus légers avec des taux de mortalité plus bas par rapport à l´adulte [3]. Une incidence pédiatrique, entre 0,8% et 2%, ont été enregistrée dans plusieurs séries à travers le monde [4,5]. Plusieurs hypothèses ont été avancées pour expliquer ça. La gravité de cette maladie chez l´enfant, mystérieusement basse, pourrait être expliquée par la vaccination active anti virale des enfants de la naissance jusqu´à cinq ans. Une réactivité croisée entre la vaccination ROR [rubéole-oreillons-rougeole] et d´autres agents viraux tels que le VIH et le Papillomavirus HPV a été démontrée [6]. Dans le domaine de la dermatologie, les verrues causées par le virus du papillome humain pourraient être améliorées en utilisant le vaccin ROR en intra-lésionnel [6]. En plus, les pays ayant un programme généralisé de vaccination par le BCG [Bacillus Calmette-Guérin] semblent avoir une incidence et un taux de mortalité par le COVID 19 inférieurs aux autres [7]. Selon Hegarty et ses collègues, l´incidence de COVID-19 est de 38,4 par million dans les pays où la vaccination BCG est pratiquée, contre 358,4 par million en l´absence de ce programme [7]. Le taux de mortalité est d´environ 4 par million dans les pays avec le programme de BCG contre 40 par million dans les pays sans de tel programme. Cela peut être dû aux avantages immunologiques connus de la vaccination par le BCG [7]. La vaccination BCG induit des changements persistants dans les cellules immunitaires innées améliorant l´immunité anti-mycobactérienne, bactérienne, fongique et virale [8]. Des témoins sains vaccinés par le BCG ont été remis en cause par le virus de la fièvre jaune et ont démontré une immunité antivirale améliorée et une charge virale réduite [7].

D´après le centre d´épidémiologie et de lutte contre les maladies, le 03 Mai 2020, le Maroc a recensé environ 5250 cas confirmés de COVID-19 dont 495 étaient des enfants de moins de 15 ans. L´incidence cumulée à l´échelle nationale était de 4,8/100 000 enfants [9], 54,3% étaient asymptomatiques et 39,7% ont présenté un tableau clinique bénin [9]. Le Maroc a lancé, précocement, une série de mesures préventives pour atténuer la transmission du coronavirus et réduire la morbi-mortalité liée à cette pandémie. Ces mesures comprennent le confinement strict à domicile, la fermeture de toutes les structures non vitales et le port obligatoire des masques à l´extérieur.

Au Maroc et ailleurs, au milieu de cette effervescence vécue sous les cieux de la COVID-19, les dispensaires de santé et les cabinets médicaux du secteur libéral connaissent une désertion inquiétante et un déclin du taux de vaccination. Il est clair que l´accès aux vaccins pour tous a transformé nos sociétés, mais c´est un outil collectivement préventif et il doit être entretenu pour être efficace, même dans les moments difficiles. L´administration des vaccins doit respecter rigoureusement le calendrier national que le Maroc a adopté, depuis des décennies, ciblant les maladies les plus graves et assurant la couverture de la majorité de la population (Tableau 1). Toute interruption de ce programme, même pendant de courtes périodes de 2 à 3 mois, entraînera une accumulation d´individus sensibles et une probabilité plus élevée de flambées épidémiques. De telles épidémies peuvent entraîner des décès et un fardeau accru pour les systèmes de santé déjà mis à rude épreuve par la réponse à la pandémie COVID-19 [10].

**Tableau 1:** le calendrier national d´immunisation des enfants de la naissance à 5 ans au Maroc

Age	Vaccins	Maladies ciblées
Durant le 1^er^ mois	- HB1- BCG- VPO (Zéro)	- Hépatite virale type B- Formes grave de la tuberculose- Poliomyélite
2 mois	- Penta 1 (DTC-Hib-HB)- VPO 1- Rotavirus 1- VPC 1	- Tétanos, Diphtérie, Coqueluche, Haemophilus Influenza type b, Hépatite virale type B- Poliomyélite- Gastroentérites graves dues au Rotavirus- Infections invasives dues au Pneumocoque
3 mois	- Penta 2 (DTC-Hib-HB)- VPO 2- Rotavirus 2	- Tétanos, Diphtérie, Coqueluche, Haemophilus Influenza type b, Hépatite virale type B- Poliomyélite- Gastroentérites graves dues au Rotavirus
4 mois	- Penta 3 (DTC-Hib-HB)- VPO 3- VPC 2	- Tétanos, Diphtérie, Coqueluche, Haemophilus Influenza type b, Hépatite virale type B- Poliomyélite- Infections invasives dues au Pneumocoque
9 mois	RR	- Rougeole et rubéole
12 mois	VPC 3	- Infections invasives à Pneumocoque
18 mois	DTC 4 VPO 4 RR	- Tétanos, Diphtérie et Coqueluche- Prévention de la poliomyélite- Rougeole et la Rubéole

En Angleterre, Le nombre de vaccinations contre la rougeole, les oreillons et la rubéole (ROR) a diminué entre Février et Avril 2020. Dans les 3 semaines suivant l´introduction des mesures de distanciation sociale, le taux de vaccination ROR a baissé de 19,8% par rapport à la même période en 2019 et la vaccination héxavalente a diminué de 6,7% avant de s´améliorer à mi-Avril après sensibilisation du grand public [11]. Aux états unis, pendant la pandémie COVID-19, le taux de vaccination ROR a baissé de 53%, celui de l´hépatite A de 51%, le HPV de 73% et la grippe en baisse de 83% [12]. Selon une autre étude américaine à Michigan, le statut vaccinal recommandé chez les nourrissons de 5 mois est passé de 67,9% en 2019 à 49,7% en Mai 2020. La couverture vaccinale contre la rougeole assurée à l´âge de 16 mois est passée de 76,1% en mai 2019 à 70% en mai 2020 [13]. Au Maroc, nous n´avons pas de chiffres officielles ou d´étude sur cette baisse mais notre enquête a révélé que la majorité des parents [82,5%] hésitent à faire le vaccin et 6% refusent carrément de venir au cabinet au cours de cette pandémie. La peur d´être contaminer au cours de la vaccination était la raison de ce comportement dans environ 70% des parents. En absence de recommandations officielles précoces, 72% des pédiatres ont décalé les rendez-vous des vaccinations de plusieurs semaines et 20% l´ont arrêté chez les enfants de plus de 18 mois. Les 2 tiers des pédiatres ont changé cette attitude au cours du 2^e^ et 3^e^ mois de la pandémie, suite au communiqué ministériel et aux vidéoconférences incitant sur la nécessité de reprendre la vaccination. L´agence des Nations Unies pour l´enfance avertit que la pandémie de coronavirus pourrait mettre en danger des millions d´enfants dans plusieurs régions du monde [14]. Selon l´UNICEF, le COVID-19 impactera sur des millions d´enfants au Moyen-Orient et en Afrique à cause de l´arrêt des vaccinations. En Irak, au Soudan, en Syrie et au Yémen, les campagnes de vaccination ont été interrompues pendant plusieurs semaines [14].

La situation risque de perdurer plusieurs mois et le retard des vaccinations contre la rougeole, la coqueluche ou les méningites à Haemophilus et à pneumocoque dans la première année pourrait avoir de graves conséquences sanitaires. En effet, au cours de l´épidémie d´Ebola de 2014-2015, le Libéria et la Guinée ont enregistré une forte baisse de plus de 25% du nombre d´enfants vaccinés contre la rougeole en 2014 et 2015. L´incidence de la rougeole a reconnu une grande hausse persistante jusqu´à deux ans après la fin de l´épidémie d´Ebola [15]. Il est primordial donc de rappeler aux parents la nécessité vitale de protéger leurs enfants contre les maladies évitables par la vaccination, même si la pandémie de COVID-19 se poursuit. Les exigences de distanciation sociale étant assouplies progressivement, les enfants qui ne sont pas protégés par des vaccins seront plus vulnérables à des maladies telles que la rougeole et la coqueluche [12]. Il faut absolument maintenir l´ensemble des vaccins obligatoires à condition de pouvoir accueillir les nourrissons dans de bonnes conditions de sécurité qui seront détaillés dans les recommandations ci-dessous. Les prestataires de services de vaccination devraient commencer à énumérer les cohortes d´enfants qui ont manqué leurs doses de vaccin et élaborer un plan d´action pour une vaccination de rattrapage sur mesure. Il faut se procurer les vaccins nécessaires avec un approvisionnement suffisant en matériels et en personnels.

La semaine mondiale de la vaccination, qui a eu lieu en fin d´avril, était une occasion pour promouvoir le recours aux vaccins et pour souligner l´importance mondiale de la vaccination pour améliorer la santé et le bien-être de tous. Au Maroc, plusieurs vidéo-conférences en ligne ou webinaires ont été réalisés pour avertir du danger de ce relâchement vaccinal et insister sur l´importance de continuer la vaccination malgré le confinement. Une large sensibilisation médiatique a été aussi renforcée afin d´expliquer aux parents la nécessité de rattraper rapidement les vaccins ratés. On estime, en revanche, que les autres vaccinations recommandées au-delà de l´âge de 2 ans peuvent être différées jusqu´à la levée des mesures de confinement. Cet avis s´appuie sur les préconisations de l´Organisation mondiale de la Santé en matière de vaccination pendant la pandémie [16] et sur la position exprimée par une majorité de sociétés savantes représentatives de la pédiatrie [17]. L´HAS [Haute Autorité Sanitaire] souligne aussi l´importance de maintenir les consultations pour vaccination, de les organiser au mieux et de respecter les mesures barrières afin de protéger les professionnels, les nourrissons et leur famille et d´éviter la transmission de virus sur les lieux de soins [18]. Nous avons élaboré, à partir des préconisations ministérielles marocaines et européennes, les recommandations suivantes sur la vaccination des enfants au cours de la pandémie COVID-19 [10,19,20]: reprendre le plus rapidement possible les consultations consacrées aux vaccinations des deux premières années de vie, en respectant les étapes prévues au calendrier vaccinal. Rattraper au plus tôt les retards de vaccination des nourrissons qui ont été accumulés. Étant donné que, les accouchements continuent à avoir lieu durant la pandémie, la vaccination des nouveau-nés [BCG, VPO, hépatite B] doit rester une priorité. Ne plus différer les rendez-vous de vaccination et d´organiser des plages de rendez-vous permettant de dissocier les consultations d´enfants malades et ceux pour vaccination. Évitez les campagnes de vaccination de masse jusqu´à la résolution de COVID-19. Retarder l´introduction de tout nouveau vaccin dans le calendrier de vaccination. Communiquer clairement à la communauté et aux professionnels de la santé l´obligation de continuer à faire la vaccination primaire. Rassurer les parents sur le fait que les mesures barrière et l´hygiène sont assurées dans les cabinets et les centres de santé: salle d´attente aérée, pas d´attente des patients, admission d´un seul parent par enfant, port de masque obligatoire, hygiène des mains, désinfection renforcée des objets et des surfaces de travail. Pour les enfants âgés de 5 ans et plus, de reporter les rendez-vous de vaccination à la fin du confinement, en maintenant les mêmes mesures barrière. Étant donné les avantages très encourageants de la vaccination, même pendant la pandémie de COVID-19, il est crucial de surveiller la tendance de l´adoption communautaire des vaccins, de rendre la vaccination des nourrissons une priorité sanitaire, et résoudre tous les obstacles possibles à la vaccination parmi le grand public et au sein des structures sanitaires.

## Conclusion

Le Maroc a lancé tôt une sensibilisation intensifiée envers les professionnels de santé et la population en ce qui concerne la vaccination durant la COVID-19. Nous avons noté, à travers notre enquête, que les pédiatres ont changé rapidement leur stratégie vaccinale en faveur du renforcement et du rattrapage afin de pallier à ce relâchement.

### Etat des connaissances sur le sujet

- La vaccination est un outil de prévention infectieuse par excellence;
- La pandémie COVID-19 a provoqué indirectement une baisse inquiétante de la vaccination des enfants partout dans le monde;
- Ce déclin observé risque d´entrainer des flambées épidémiques de maladies infectieuses.

### Contribution de notre étude à la connaissance

- Notre étude confirme que la vaccination a subi une diminution alarmante, essentiellement secondaire à la peur de contamination et aux difficultés d´accès aux structures sanitaires liées au confinement obligatoire ;
- Nous avons élaboré, à travers des sociétés savantes, des recommandations vaccinales claires au cours de la pandémie COVID-19.
